# The role of self‐compassion in the mental health of adults with ADHD

**DOI:** 10.1002/jclp.23354

**Published:** 2022-03-25

**Authors:** Danielle M. Beaton, Fuschia Sirois, Elizabeth Milne

**Affiliations:** ^1^ Department of Psychology University of Sheffield Sheffield UK

**Keywords:** ADHD, mental health, self‐compassion, structural equation modelling, well‐being

## Abstract

**Objective:**

Evidence suggests that the poorer mental health associated with attention deficit hyperactivity Disorder (ADHD) is partially explained by adverse psychosocial correlates of the condition. As recent studies show that self‐compassion is negatively associated with ADHD, this study investigates if levels of self‐compassion may explain the mental health outcomes in people with ADHD compared to people without ADHD.

**Method:**

A total of 543 adults with ADHD (62.72% female, 18–67 years), and 313 adults without ADHD (66.45% female, 18–82 years) completed questionnaires online to measure levels of self‐compassion and mental health. A Structural Equation Model assessed the mediating effect of self‐compassion on the relationships between ADHD and well‐being (psychological, emotional, and social), and ADHD and ill‐being (depression, anxiety, and stress).

**Results:**

Findings suggest that low self‐compassion contributes to poorer mental health in adults with ADHD compared to adults without ADHD.

**Conclusions:**

Thus, self‐compassion may be a potential target to improve mental health in this population.

**Public Health Significance:**

This study shows that self‐compassion is an important factor in the mental health of adults with ADHD and provides preliminary evidence for the use of self‐compassion interventions to improve mental health outcomes in adults with ADHD.

## 1 INTRODUCTION

Attention deficit hyperactivity Disorder (ADHD) is a highly prevalent disorder, whereby an estimated 3.4% of adults (aged 18–44) globally are estimated to have ADHD (Fayyad et al., 2017). Retrospective clinical studies report that up to 50% of people with clinical ADHD or high ADHD traits experience depressive episodes or increased anxiety (Biederman et al., 1993; Kessler et al., 2006; Ollendick et al., 2008). People with ADHD also have a lower tolerance for stress, labile moods, and higher levels of perceived stress compared to individuals without ADHD (Combs et al., 2015; Miklósi et al., 2016; Yeguez et al., 2018). Moreover, people with ADHD report lower levels of life satisfaction, quality of life, and overall well‐being (Agarwal et al., 2012; Buchanan, 2011; Gudjonsson et al., 2009; Ogg et al., 2016). Research conducted to explain why people with ADHD experience reduced mental health has mostly focused on biological factors (see, Andersson et al., 2020, for a review). However, the notion that psychosocial problems associated with ADHD increase the risk of negative mental health outcomes in people with ADHD is gaining popularity (Roy et al., 2015; Schatz & Rostain, 2006; Simmons & Antshel, 2020). This theory suggests that ADHD creates a more negative environment, characterized by experiences of failure, rejection, and stress, that makes it more difficult for people with ADHD to flourish and manage their mental health (Powell et al., 2020, 2021; Roy et al., 2015; Schatz & Rostain, 2006; Simmons & Antshel, 2020). Other emerging evidence suggests that self‐compassion may be particularly important for the mental health of adults with ADHD (Geurts et al., 2020). Therefore, understanding the potential role of self‐compassion for the well‐being and ill‐being of adults with ADHD is an important aim that can inform interventions targeted at improving mental health in this population.

Self‐compassion is a healthy way of relating to oneself during times of emotional or physical suffering (Neff, 2003a). Neff (2003b) operationalizes self‐compassion based on three core elements. Each element balances responses toward the self during times of difficulty along a spectrum between (1) self‐kindness and self‐judgment (being kind and understanding vs. being critical and judgemental); (2) mindfulness and overidentification (taking a mindful, balanced stance toward thoughts and feelings vs. becoming consumed by negative reactivity); and (3) common humanity and isolation (acknowledging that suffering is experienced by all humans vs. the belief that the suffering is isolated to oneself). To be high in self‐compassion is to show kindness and understanding toward the self in times of suffering, similarly to how a person would show compassion toward others (Neff, 2003a). Thus, when an individual responds with self‐compassion, they recognize that all people have negative experiences, can take a balanced view of these experiences, and treat themselves with acceptance and kindness, without being judgemental, feeling like bad things only happen to them, or becoming over‐identified with the negative feelings they experience.

Emerging research indicates that self‐compassion is significantly lower in people with ADHD. Willoughby and Evans (2019) classified undergraduate students with ADHD and/or a learning disorder by levels of self‐compassion and found that more of the sample had categorically low levels of self‐compassion compared to moderate or high levels. Moreover, Beaton et al. (2020) compared levels of self‐compassion between adults with and without ADHD and found that adults with high traits of ADHD had significantly lower levels of self‐compassion irrespective of their diagnostic status and of co‐occurring mood conditions. There is also early evidence that self‐compassion may be an important factor for improving well‐being in adults with ADHD. A recent study investigating the effect of mindfulness‐based cognitive therapy (MBCT) in adults with ADHD reported that improvements in well‐being were explained by increases in self‐compassion (Geurts et al., 2020). Well‐being in this study followed the definition by Keyes (2002), which proposes that well‐being can be measured on a scale from languishing to flourishing through levels of emotional, psychological, and social well‐being. The results from Geurts et al. (2020) suggest that self‐compassion may be an important factor in the overall well‐being of adults with ADHD.

In neurotypical populations, levels of self‐compassion are a strong predictor of both well‐being and ill‐being (MacBeth & Gumley, 2012; Zessin et al., 2015). In fact, high self‐compassion predicts higher levels of well‐being above other factors that have well‐established associations with well‐being such as goal regulation, social support, and stress (Neely et al., 2009). In contrast, low self‐compassion predicts levels of depression, anxiety, and high stress even when controlling for variables known to contribute to such factors, including self‐criticism and self‐esteem (Neff et al., 2005, 2007; Raes, 2011). Moreover, a meta‐analysis of 21 randomized control trials demonstrated that self‐compassion interventions are predictive of significant increases in well‐being and decreases in ill‐being (Kirby et al., 2017). If self‐compassion contributes to the mental health of neurotypical individuals, it stands that self‐compassion may be a contributing factor for the mental health of adults with ADHD.

Furthermore, a recent study has demonstrated self‐compassion may be an important contributing factor to the mental health of neurodiverse populations. Galvin et al. (2021) demonstrated that self‐compassion significantly mediated the relationships between levels of autistic traits and depression and anxiety in a sample of 164 university students. Although this study was conducted with a relatively small nonclinical student sample, the results support the possibility that self‐compassion may be an important factor for consideration in neurodiverse populations and, therefore, warrants further investigation.

Given the protective value of self‐compassion, it is particularly important to assess self‐compassion in people with ADHD because they are likely to experience more negative events (Asherson et al., 2018) and more stressful daily and life events that negatively impact mental health (Friedrichs et al., 2012; Skirrow et al., 2014). For example, ADHD is associated with poor academic outcomes (Loe & Feldman, 2007), impaired relationships (Murphy & Barkley, 1996), and increased stigma, criticism, and rejection (Canu et al., 2008; Hoza, 2007; Psychogiou et al., 2007), and each of these factors have been shown to explain the link between ADHD and levels of depression (Powell et al., 2020, 2021; Simmons & Antshel, 2020). In neurotypical populations, self‐compassion has been shown to buffer the negative emotional consequences of academic failures (Neff et al., 2005), relationship issues (Sbarra et al., 2012; Yarnell & Neff, 2013), and stigma/criticism (Neff & Faso, 2015; Vigna et al., 2018; Wong et al., 2016). This has been demonstrated in a longitudinal study that assessed levels of self‐compassion and mental health as high school students transitioned to university (Kroshus et al., 2021). Levels of depression and anxiety increased as a result of the stressful transition, however, those with lower levels of self‐compassion had worse mental health outcomes compared to those with higher levels of self‐compassion.

Self‐compassion does not reduce negative experiences or emotions, but it is thought to alter the way that people relate to negative events—buffering the effects of negative experiences on mental health (Zessin et al., 2015). Hence, it is reasonable to expect that if the mental health of people with ADHD is affected by the more frequent and numerous negative experiences as a consequence of the condition (Asherson et al., 2018), then the increased tendency to relate to themselves with low self‐compassion during these difficult times (Beaton et al., 2020; Willoughby & Evans, 2019) may contribute to poor mental health.

Self‐compassion also has commonalities with many of the individual differences that are known to contribute to the mental health of people with ADHD. For example, emotion dysregulation (Bodalski et al., 2019), maladaptive self‐schemata (Miklósi et al., 2016), and limited resilience (Regalla et al., 2015) are factors that have been implicated in the mental health of people with ADHD (Knouse et al., 2008; Oddo et al., 2018). These factors also reflect low self‐compassion. Self‐compassion is an adaptive self‐schema (Neff, 2003a), it is also a resilience factor during times of failure and stress, helping people cope with long‐term challenges (Neff & Faso, 2015; Sirois et al., 2015), and facilitating the use of healthy emotion regulation strategies (Finlay‐Jones et al., 2015). Therefore, self‐compassion is a single factor that represents many of the elements considered as potential underlying factors in the mental health of people with ADHD.

The two‐continua model of mental health (Keyes et al., 2002) acknowledges that both well‐being and ill‐being contribute to a person's overall mental health, but that they can exist independently. This model proposes that well‐being and ill‐being do not exist on a linear continuum but that they exist on a dual‐axis (Keyes et al., 2002). Keyes and colleagues have conducted a series of studies to demonstrate that people can have ill‐being symptoms—such as depression—but still flourish in their well‐being, and vice versa, people can have symptoms of languishing in their well‐being but have no symptoms of ill‐being (Keyes, 2005, 2006; Keyes et al., 2008; Lamers et al., 2010). Therefore, it is important to consider both well‐being and ill‐being in the same model when investigating mental health. Geurts et al. (2020) study suggests that self‐compassion is a contributing factor for well‐being in adults with ADHD, however, the study did not report if self‐compassion was associated with measures of ill‐being. Moreover, the study also did not address whether self‐compassion explains differences in mental health between adults with and without ADHD, and therefore, it is still unknown whether levels of self‐compassion explain the discrepancy in mental health between those with and without the condition.

Self‐compassion is known to be lower in adults with ADHD (Beaton et al., 2020), but it is still unknown if self‐compassion contributes to the overall mental health of adults with ADHD. Accordingly, the current study aims to investigate the role that self‐compassion may have in explaining differences in ill‐being and well‐being between people with and without ADHD. It was hypothesized that lower levels of self‐compassion in adults with a self‐reported diagnosis of ADHD would mediate the positive relationship between ADHD status and ill‐being (depression, anxiety, and stress), and a negative relationship between ADHD status and well‐being (psychological, emotional, and social).

## 2 METHOD

### 2.1 Participants

Participants were recruited between January and March 2019, via social media sites, online forums, posters displayed in shops, university disability services, and at ADHD support group venues across the United Kingdom. Data collected from this study have been partially presented previously (Beaton et al., 2020). In total, 1589 participants originally took part in the study, 283 participants data were excluded from analysis because the data were incomplete, and 66 participants were excluded because they did not pass the validity check question. An ADHD screening questionnaire (adult ADHD Self‐report Scale‐V1.1 [ASRS‐V1.1] Kessler et al., 2005) was used to confirm that participants symptoms of ADHD were highly consistent with an adult ADHD diagnosis, and that participants in the No‐ADHD group did not meet the clinical threshold. This was to ensure that the sample was fully representative of people “with” and “without” ADHD irrespective of diagnostic status. Consequently, 30 participants in the ADHD group were excluded because their trait scores did not align with their self‐reported diagnostic status. In the No‐ADHD group, 354 participants were excluded because they scored over the threshold on the screening questionnaire. The data regarding levels of self‐compassion in participants with high ADHD traits and no diagnosis are reported in Beaton et al., (2020). The final analytic sample included 856 participants: 543 participants with a self‐reported diagnosis and high traits of ADHD and 313 participants without a self‐reported diagnosis and low traits of ADHD. A minimum desired sample size of 500 was required, based on the general rule that 1:10 participants per parameter are required to conduct structural equation modeling (SEM) (Bentler & Chou, 1987; Kline, 2012), therefore, even after data exclusion the sample was sufficient for analysis. The ADHD group were aged between 18 and 67 years old (*M* = 33.56, SD = 10.43), 88.58% were Caucasian, and 63.72% were female. The No‐ADHD group was aged between 18 and 82 years old (*M* = 33.83, SD = 11.93), 84.35% were Caucasian, and 66.45% were female. Further details of the sample's demographics are displayed in Table 1.

**Table 1:** Sociodemographic and clinical profile of the ADHD and No‐ADHD groups

	ADHD (*n* = 543)	No ADHD (*n* = 313)
Employment status, *n* (%)		
Employed	379 (69.79)	235 (75.10)
Unemployed	124 (22.84)	61 (19.49)
Disabled/sickness leave	37 (6.81)	8 (2.56)
Retired	3 (<1)	9 (2.88)
Student, *n* (%)	169 (31.12)	97 (30.99)
Current place of residence, *n* (%)		
United Kingdom	303 (55.80)	271 (86.58)
United States	155 (28.55)	14 (4.79)
Other	85 (15.63)	28 (8.95)
Relationship status, *n* (%)		
Single	164 (30.20)	83 (25.88)
Married/cohabiting/in relationship	341 (62.80)	224 (71.57)
Separated/divorced/widowed	38 (7.00)	6 (1.92)
Highest level of education, *n* (%)		
Less than secondary school	9 (1.66)	1 (>1.00)
Secondary school	59 (10.87)	35 (11.18)
College/sixth form	217 (39.96)	83 (27.16)
University degree	163 (30.02)	102 (32.59)
Postgraduate/professional degree	95 (17.50)	92 (29.39)
Self‐reported clinical diagnosis, *n* (%)		
Behavioral disorder	19 (3.45)	0
Mood disorder	296 (54.51)	66 (21.09)
Autism spectrum disorder	44 (8.10)	4 (1.28)
Obsessive‐compulsive disorder	25 (4.60)	8 (2.56)

### 2.2 Procedure

Participants completed a series of questionnaires online via the survey platform Qualtrics (www.qualtrics.com). Demographic questions were presented first and were always followed by measures of ADHD and self‐compassion. Measures of stress, well‐being, perceived criticism,^1^ and an attention check question were then presented in random order. The measure of depression and anxiety was always presented last, to reduce the possibility of negative priming.

### 2.3 Measures

#### 2.3.1 ASRS‐V1.1

The ASRS‐V1.1 (Kessler et al., 2005) self‐report questionnaire is a widely used screener for ADHD. It includes six items written following the DSM‐IV diagnostic criteria of ADHD and reflects ADHD symptoms in adults. The frequency of symptoms is rated on a five‐point Likert‐type scale from 0 (never) to 4 (always). One point is given for any answers greater than 2 for the first three items, or any answers greater than 3 for the final three items. A sum of four or more ADHD symptoms, on a possible range of 0–6, is considered as highly consistent with an ADHD diagnosis in adults (Adler et al., 2006; Kessler et al., 2007). Previous studies report high sensitivity and moderate positive predictive power and specificity, which indicates that the ASRS successfully identifies someone with ADHD and would rarely identify someone with ADHD incorrectly (Hines et al., 2012). In this study, internal consistency (*α* = 0.87 and *ω* = 0.87) was similar to previous studies (*α* = 0.84; Kessler et al., 2006).

#### 2.3.2 The Self‐Compassion Scale (SCS)

The SCS (Neff, 2003b) is a 26‐item self‐report questionnaire that consists of six subscales: self‐kindness, that is, “I try to be understanding and patient towards those aspects of my personality I don't like”; self‐judgment, for example, “I'm disapproving and judgmental about my own flaws and inadequacies”; common humanity, for example, “I try to see my failings as part of the human condition”; isolation, for example, “When I think about my inadequacies it tends to make me feel more separate and cut off from the rest of the world”; mindfulness, for example, “When something painful happens I try to take a balanced view of the situation”; and overidentification, for example, “When I'm feeling down, I tend to obsess and fixate on everything that's wrong.” Typically, the items in the SCS are rated on a scale of 1–5. However, to improve the discrimination and reliability of the scale (Chomeya, 2010), in this study, items were rated on a scale of 1 (almost never) to 6 (almost always). A six‐point scale encourages participants to lean more toward a positive or negative response rather than providing a neutral response, which can be interpreted differently between respondents (Youn et al., 2017). Having more response options does not impact means, standard deviations, or correlations, but it does reduce the possibility of skewness and increase scale sensitivity (Leung, 2011), and ensures there is no misinterpretation over what the neutral midpoint means (Youn et al., 2017). Higher levels of self‐compassion are reflected in high scores for self‐kindness, common humanity, and mindfulness and low scores for self‐judgment, isolation, and overidentification. A global self‐compassion score is calculated by reverse coding the negative items, summing all six‐subscale scores, and dividing by six to attain the mean value. When using a five‐point Likert scale, the scale for global self‐compassion has high levels of internal consistency (*α* = 0.92) (Mantelou & Karakasidou, 2017; Neff, 2003b), and high test–retest reliability (*α* = 0.93) (Neff et al., 2005; Neff, 2003b). In this study global self‐compassion had comparable levels of internal consistency (*α* = 0.94 and *ω* = 0.94).

#### 2.3.3 The Mental Health Continuum: Short Form (MHC‐SF)

The MHC‐SF (Keyes, 2009) is a self‐report questionnaire that includes 14 items across three subscales. Three items represent feelings of emotional well‐being, six items represent feelings of psychological well‐being, and five items represent feelings of social well‐being. Psychological well‐being represents the psychological aspects of eudaimonic well‐being and focuses on optimal functioning in terms of individual fulfillment, while social well‐being represents the societal aspects of eudaimonic well‐being, indicating how optimally a person is functioning in society. Emotional well‐being involves feelings of happiness, satisfaction, and interest in life, representing the hedonic element of well‐being. Respondents rate how frequently they have experienced each item over the previous month, on a six‐point Likert scale ranging from never (0) to every day (5). Individual scores for each subscale are calculated by summing the responses for the corresponding sub‐scales. As such, emotional well‐being scores range between 0 and 15; social well‐being scores range between 0 and 25, and psychological well‐being scores range between 0 and 30. The MHC‐SF has adequate to high internal consistency across each of the subscales: emotional well‐being (*α* = 0.83) psychological well‐being (*α* = 0.83), and social well‐being (*α* = 0.74) (Lamers et al., 2010). Similar levels of internal consistency were also seen in this current study: emotional well‐being (*α* = 0.86, *ω* = 0.86), psychological well‐being (*α* = 0.84, *ω* = 0.84), and social well‐being (*α* = 0.76, *ω* = 0.76).

#### 2.3.4 The Hospital Anxiety and Depression Scale (HADS)

The HADS (Zigmond & Snaith, 1983) is a 14 item self‐report questionnaire that contains two subscales: depression (seven items) and anxiety (seven items). HADS‐depression focuses on anhedonia, a core symptom of depression, and HADS‐anxiety focuses on generalized anxiety disorder symptoms. Each item is scored on a response scale with four alternatives ranging between 0 and 3. A total score between 0 and 21 can be calculated for each subscale by reverse scoring six items and summing all responses. The concurrent validity of the HADS ranges from good‐to‐very good (0.60–0.80) and has similar levels of sensitivity and specificity (0.80) to the General Health Questionnaire (Bjelland et al., 2002). Previous research has reported high internal consistency for the HADS‐depression (*α* = 0.71) and HADS‐anxiety (*α* = 0.84) when used with an ADHD population (Grogan & Bramham, 2016) and in this study, internal consistency was also high for the HADS‐depression (*α* = 0.83 and *ω* = 0.84), and adequate internal consistency for HADS‐anxiety (*α* = 0.76 and *ω* = 0.77).

#### 2.3.5 The Perceived Stress Scale (PSS)

The PSS (Cohen et al., 1983) is a 10‐item questionnaire that includes four positively worded and six negatively worded items that represent how unpredictable, uncontrollable and overloaded respondents may find their lives. The frequency at which respondents identify with each item is scored on a five‐point Likert scale from never (0) to very often (4). Positively worded items are reverse scored, and all item responses are summed to calculate an individual score ranging between 0 and 40. Higher scores indicate higher levels of perceived stress. The PSS has a good level of internal consistency (*α* > 0.70) and test–retest reliability (intraclass correlation > 0.70) (Lee, 2012). In this study, PSS showed high levels of internal consistency (*α* = 0.89 and *ω* = 0.89).

#### 2.3.6 Attention check

An attention check question was included to ensure that respondents were attending to the questions and responding accurately. The question read: “People vary in the amount they pay attention to these kinds of surveys. Some take them seriously and read each question, whereas others go very quickly, and barely read the questions at all. If you have read this question carefully, please write the word yes in the blank box below labelled 'other'. There is no need for you to respond to the scale below.” Respondents were required to respond in an empty text box labeled “Other” to pass the attention check.

### 2.4 Data analysis

The data were cleaned, coded, and analyzed in Excel and R (R Development Core Team, 2013). The data were mean centered and rounded to two decimal places. Confidence intervals reported are 95%. Assumptions of normality were assessed through QQ‐plots and density plot histograms, and scatterplots were used to assess homoscedasticity and autocorrelation of residuals. Multicollinearity was assessed via a correlation matrix using Pearson's R and variance inflation factors (VIF). Data points were considered outliers if they were 1.5 times greater than the interquartile range (IQR) of the group and caused changes to the assumptions or model relationships.

SEM was used to model the mediating effect of self‐compassion on the relationship between ADHD (IV) and two latent variables, ill‐being (depression, anxiety, stress), and well‐being (psychological, emotional, and social well‐being). Each of the dependent variables (depression, anxiety, stress, psychological, emotional, and social well‐being) were also loaded onto a global latent factor of “Mental Health” to control for any shared variance across variables. This structure was theoretically driven by the two continua model (Keyes et al., 2002). SEM is a powerful form of multivariate analysis, which uses a combination of factor analysis and regression/path analysis, to determine the structural relationship between measured variables and latent constructs. It allows the observation of interrelated dependence of variables in a single analysis and heightens the ability to draw more causal conclusions (Elliot, 2003). The SEM was conducted following four steps: (1) Assumptions for the model were tested, the data were mean centered, and the model was stated using path‐analysis. (2) A factor analysis was conducted to ensure the proposed latent variables were accurately represented by the observed variables (see Supporting Information: Materials 1). (3) A bivariate SEM model was fitted. (4) The fit of the model was assessed, and any modifications made were based on the modification indices suggested by statistical software, and on the researcher's intuition. Following the recommendation Kline (2012), a good model fit will be assessed through the following criteria: *χ* ^2^ (*p* < 0.05), root mean square error approximation (RMSEA) (<0.08), standardised root mean square residual (SRMR) (<0.08), comparitive fit index (CFI) (≥0.90), and Tucker‐Lewis index (TLI) (≥0.90).

The analysis presented in this current study was pre‐registered on the Open Science Framework. Although the analysis was pre‐registered, the model used for the SEM analysis deviates from the preregistered model because CFA analysis confirmed that it was not the best representation of the data (Supporting Information: Materials 1). Moreover, in hindsight, it was theoretically more accurate to use a bivariate model that accounted for shared variance across the different measures of mental health. The correlational analysis also indicated a need to control for comorbidity and gender.

## 3 RESULTS

### 3.1 Descriptive statistics

Mardia tests revealed significant multivariate skew (*M* = 314.03, *p* < 0.000), thus, robust maximum likelihood estimators were used for the SEM, as these estimators do not require assumptions of normality (Finney & DiStefano, 2008). The descriptive statistics for each variable are shown in Table 2, the Pearson's *R* correlations for the whole sample are presented in Table 3.

**Table 2:** Mean and standard deviations of each measure before mean centering, by ADHD group

	ADHD		No‐ADHD		
	*M*	SD	*M*	SD	*t test*
Self‐compassion	2.57	0.76	3.35	0.87	13.69*
Psychological well being	13.40	6.24	18.24	6.70	10.55*
Emotional well being	8.06	3.43	9.80	3.41	7.16*
Social well being	8.62	5.15	11.21	5.50	6.93*
Depression	8.18	4.09	5.70	3.96	−8.63*
Anxiety	12.72	4.00	8.55	4.48	−14.03*
Perceived stress	27.71	5.23	21.71	5.99	−15.33*

*Note*: Between group differences assessed using individual *t* tests.

* *p* < 0.01.

**Table 3:** Pearson's *R* correlations between measures

	1	2	3	4	5	6	7
1. ADHD ASRS score							
2. Self‐compassion	−0.52						
3. Psychological well being	−0.41	0.64					
4. Emotional well being	−0.28	0.54	0.70				
5. Social well being	−0.30	0.55	0.67	0.62			
6. Depression	0.33	−0.53	−0.62	−0.71	−0.51		
7. Anxiety	0.54	−0.60	−0.51	−0.47	−0.43	0.52	
8. Perceived stress	0.56	−0.65	−0.56	−0.53	−0.46	0.58	0.70

*Note*: All correlations were significant to *p* < 0.01.

### 3.2 SEM analysis

**Figure 1:**
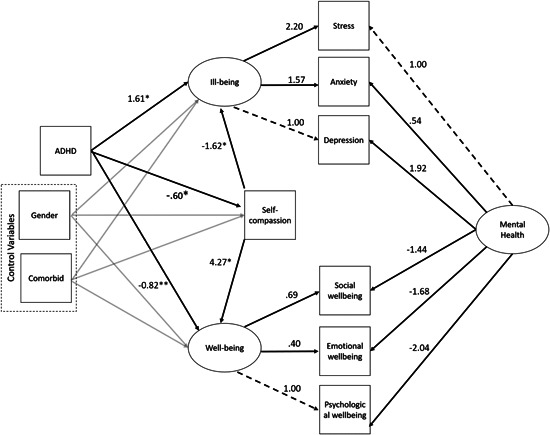
SEM model of the relationships between ADHD, well‐being, and ill‐being. Unstandardized coefficients demonstrate the direct relationships between manifest and latent variables

The model presented in Figure 1, controlling for gender and comorbidity, showed good fit to the data (*χ* ^2^ = 120.51, *df* = 20, *p* < 0.000, RMSEA = 0.08 [0.06, 0.09], SRMR = 0.04, CFI = 0.98, TLI = 0.95). Modification indices were assessed but no changes were made to the model. The linear relationships between variables in the model are presented in Figure 1.

The results from the SEM demonstrated that having a diagnosis of ADHD was significantly associated with lower self‐compassion levels (*β *= −0.6, *p* < 0.01, [−0.71 to −0.48]), lower well‐being (total effect: *β* = −3.38, *p* < 0.01, [−4.29 to −2.47]), and higher ill‐being (total effect: *β* = 2.13, *p* < 0.01, [1.72–2.54]). Although having a diagnosis of ADHD still had a significant direct effect on mental health after self‐compassion was entered into the model as a mediator (ill‐being: *β* = 1.16, *p* < 0.01, [0.85 to –1.47], well‐being: *β *= −0.82, *p* < 0.05, [−1.60 to −0.04]), levels of self‐compassion did partially mediate the relationship between having an ADHD diagnosis and increased ill‐being (indirect effect: *β *= 1.00, *p* < 0.01, [0.71–1.23]) as well as partially mediate the relationship between having ADHD and decreased well‐being (indirect effect: *β* = −2.56, *p* < 0.01, [−3.13 to −1.99]). Gender was not significantly associated with well‐being (*β *= −0.09, *p* = 0.78, [−0.71 to 0.53]) or ill‐being (*β* = −0.22, *p* = 0.07, [−0.45 to 0.02]), however, having a comorbid condition was associated with well‐being (*β* = −0.82, *p* < 0.05, [−1.60 to −0.04]), ill‐being (*β *= 0.72, *p* < 0.01, [0.43–1.02]), and self‐compassion (*β* =  −0.48, *p* < 0.05, [−0.59 to −0.37]).

## 4 DISCUSSION

This study investigated if low levels of self‐compassion contribute to the poorer mental health commonly observed in adults with ADHD. In partial accordance with our hypotheses, lower levels of self‐compassion in adults with ADHD, partially explain the higher levels of ill‐being (depression, anxiety, and stress) and lower levels of well‐being (psychological, emotional, and social well‐being) associated with ADHD. This is the first study to provide evidence that the way in which adults with ADHD respond to themselves during times of suffering or failure partially explains the poorer mental health that they have in comparison to adults without ADHD. Consequently, the findings support and extend theory and knowledge about the mental health of adults with ADHD in several important ways, and highlight potential avenues for future research. The findings also highlight the potential of self‐compassion as a positive addition to clinical practice that could complement other empirical interventions to improve self‐regulation, resilience, and mental health in adults with ADHD.

First, the current findings add to the growing evidence base that considers individual differences in adults with ADHD, and how these may interact with the outcomes of ADHD to impact factors of mental health. The results of this study demonstrate that levels of psychological, emotional, and social well‐being are all lower in adults with a diagnosis of ADHD compared to adults without ADHD. This is the first study that we know of, to show that all elements of wellbeing are reduced in people with ADHD and that both ill‐being and well‐being differ significantly in people with ADHD compared to people without ADHD. What is more, the results identified that lower levels of self‐compassion associated with ADHD were a partial contributor to both the lower well‐being and higher ill‐being observed in the participants with ADHD. This is consistent with the findings of Geurts et al. (2020) who found that improvements in the well‐being of adults with ADHD following 8‐weeks of MBCT were explained by increases in self‐compassion. Taken together, this study suggests that self‐compassion is a contributing factor to the overall mental health of adults with ADHD and highlights the potential benefit that self‐compassion interventions may have for mental health in adults with the condition. The results of the current study are consistent with evidence that self‐compassion mediates the relationship between ASD traits and ill‐being (Galvin et al., 2021). This supports the proposition that self‐compassion may be important for understanding and treating ill‐being in neurodiverse adults. Both studies demonstrate that levels of self‐compassion account for part of the association between traits that represent neurodiversity and levels of depression and anxiety. This identifies that self‐compassion could act as a potential target for clinical intervention not only in adults with ADHD but for multiple sub‐groups of people who show traits of high neurodiversity and face similar levels of daily adversity and challenge (Howlin & Magiati, 2017).

People with ADHD, and traits of neurodiversity, are more likely to experience failure (Fredriksen et al., 2014), rejection and stigma (Canu et al., 2008), and stressful daily and life events (Almeida, 2005; Semeijn et al., 2015). This study expands on the theory that adverse psychosocial factors may contribute toward the poor mental health in people with ADHD (Roy et al., 2015; Schatz & Rostain, 2006), by showing that how people with ADHD respond in times of suffering or failure is also a contributing factor. This is of particular importance because it indicates that the mental health of people with ADHD does not only rely on reducing symptoms of the condition to improve psychosocial outcomes but that modifying how people with ADHD respond to adverse situations associated with the condition may also be beneficial. Self‐compassion buffers the negative emotional responses to adverse life events which ultimately protects mental health outcomes (Kroshus et al., 2021; Vigna et al., 2018). Therefore, this study identifies a potential mechanism to protect against the negative effects that psychosocial adversities linked to ADHD can have on the mental health of people with traits of the condition (Piek et al., 2007).

Overall, these results highlight the potential self‐compassion could have for promoting well‐being in adults with ADHD. ADHD is a complex condition that is highly heterogeneous and can differ significantly from person to person (Faraone et al., 2015). In turn, this can make treatment of ADHD and co‐occurring conditions equally complex (Asherson et al., 2018). As the results of this study have identified that low levels of self‐compassion are a contributing factor for both ill‐being and well‐being in people with ADHD, self‐compassion may be a welcome clinical intervention that could benefit all adults with ADHD alongside other empirically tested interventions. To protect and improve overall mental health, increasing levels of self‐compassion could be used to support resilience and coping for those with ADHD who face challenges as a result of executive functioning deficits, but who have no diagnosed co‐occurring conditions of ill‐being. Increasing levels of self‐compassion could also be used to improve symptoms of ill‐being in the 50% of people with ADHD who have existing co‐occurring depression or anxiety (Biederman et al., 1993; Kessler et al., 2006; Ollendick et al., 2008). Although self‐compassion can be viewed as a trait‐like quality (Neff, 2003a), it can be cultivated through formal group‐based interventions such as Compassion‐Focused Therapy (Gilbert, 2009) and the Mindful Self‐Compassion program (Germer & Neff, 2013), or can be trained at home through daily practice (Galante et al., 2014) and writing exercises (Smeets et al., 2014). Consequently, future research should consider investigating the effects of increasing self‐compassion in adults with ADHD on global mental health through a longitudinal intervention‐based study.

### 4.1 Strengths and limitations

A notable strength of the current study is the large sample size, which permitted advanced statistical analysis and allowed us to be more stringent with our exclusion criteria by screening participants based on their ASRS scores. This meant that we could clearly define the ADHD and No‐ADHD groups by diagnosis and trait level and that the sample was diverse, so the findings are both representative and generalizable. The full sample included people from a range of ages, demographics, and ethnicities, and the ADHD sample was representative of the commonly co‐occurring conditions, and of people who received a diagnosis in childhood and adulthood. However, the study relied on self‐reported measures, which may make the data vulnerable to common method bias which occurs when similarity in response style, social desirability, and priming effects result in spurious correlations between items (Podsakoff et al., 2003). In turn, the cross‐sectional design prevents any conclusions about causality. Although SEM heightens the ability to draw more causal conclusions than traditional regression analysis, the findings do not confirm that a change in one variable, for example, self‐compassion, precedes a change in another (Elliot, 2003). However, the results were in line with theory and previous longitudinal evidence that self‐compassion predicts changes in well‐being (Geurts et al., 2020)—supporting the assumed temporal precedence of self‐compassion on mental health. Finally, this study used a six‐point scale instead of the traditional five‐point scale to measure self‐compassion. It has been debated that six‐points have better discrimination and reliability over the five‐point scale (Chomeya, 2010), however, this does mean that we cannot reliably compare values of self‐compassion in this study with the values of other studies that utilized the five‐point scale.

## 5 CONCLUSIONS

The current study suggests that lower levels of self‐compassion in adults with ADHD partially explain why adults with ADHD have lower levels of psychological, social, and emotional well‐being and higher levels of depression, anxiety, and stress compared to adults without ADHD. Longitudinal research is needed to verify if increasing self‐compassion can improve mental health outcomes associated with ADHD. Nevertheless, the current findings demonstrate that self‐compassion may be an important factor in many different aspects of mental health for people with ADHD. It, therefore, provides preliminary support for the use of self‐compassion interventions in adults with ADHD, in conjunction with existing empirically supported interventions.

### PEER REVIEW

The peer review history for this article is available at https://publons.com/publon/10.1002/jclp.23354

## Supporting information

Supporting information.

## Data Availability

The data that support the findings will be available in the open science framework at https://osf.io/uwekq following an embargo from the date of publication to allow for the commercialization of research findings.

## ENDNOTE

1 Perceived criticism (Hooley & Teasdale, 1989) was not included in the analysis of this current study but has been reported in Beaton et al. (2020).
