# Supplementary material for: The role of self‐compassion in the mental health of adults with ADHD
Source: J Clin Psychol. 2022 Mar 25;78(12):2497–512. doi: 10.1002/jclp.23354 (PMC9790285; doi:10.1002/jclp.23354)
Supplement: Supplementary file 1 — Supporting information. [file JCLP-78-2497-s001.docx]

Supplementary Materials 1

# Confirmatory Factor Analysis - Model Comparisons.

### Data Analysis

Confirmatory Factor Analsyis (CFA) was performed on one half of the complete dataset (n = 619). The data comprised of depression and anxiety scores, perceived stress scores, and scores of psychological, emotional and social well-being from 619 participants (65% female). All of the data was mean centered prior to analysis. The assumption of multivariate normality was not achieved as Mardia Tests revealed there was significant multivariate skew (M = 138.75, p < .000), although kurtosis was non-significant (M = 1.70, p = .09). To account for this, Robust Maximum Liklihood estimators were used to run the analysis.

### Results

#### Table 1.

##### Statistics for model of best fit, using centered, unstandardised data.

|  | Baseline Model | Model 1 | Model 2 | Model 2 |
| --- | --- | --- | --- | --- |
| Chi-square(df) | 1708.21 (15) | 189.97 (9) | 475.0 (9) | 14.12 (3) |
| CFI |  | .89 | .74 | .99 |
| TLI |  | .82 | .57 | .97 |
| RMSEA |  | .19 | .30 | .09 |
| SRMR |  | .07 | .34 | .02 |

The first model (see Figure 1) entered all dependent variables (depression, anxiety, stress, psychological well-being, emotional well-being, and social well-being) onto one factor of mental health. As can be seen in Table 1, the Confirmatory Factor Index (CFI) and Tucker Lewis Index (TLI) were high, but did not meet the recommended threshold of >.90. The Root Mean Square Error of Approximation (RMSEA), was higher than the recommended threshold of <.05. Overall, this suggests that this model is not the best fit for this data.

#### Figure 1.

##### Results of the CFA analysis on model 1.


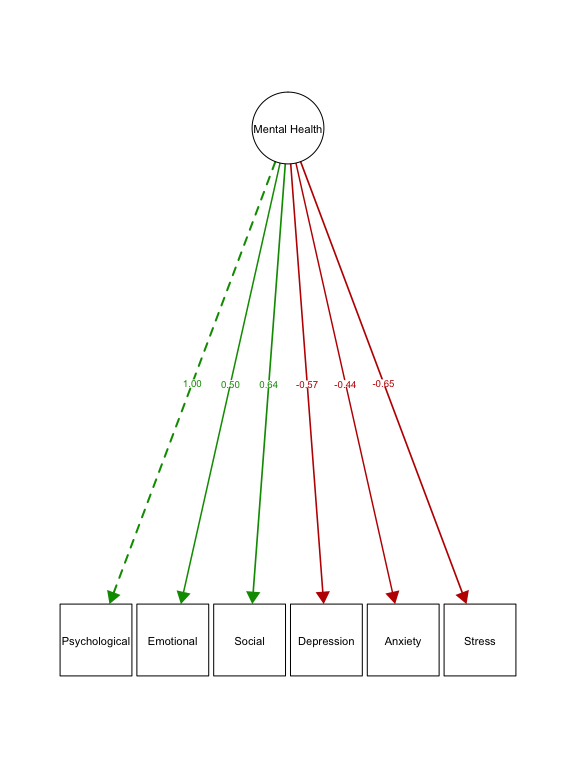


The second model was originally proposed during pre-registration. This model (see Figure 2) assumed that depression, anxiety and stress would load onto one factor (ill-being), and psychological wellbeing, social wellbeing, and emotional wellbeing would load onto a second factor (well-being). This model had poor fit to the data, with a low CFI and TFI, and high RMSEA and SRMR (see Table 1).

#### Figure 2.

##### Results of the CFA analysis on model 2.


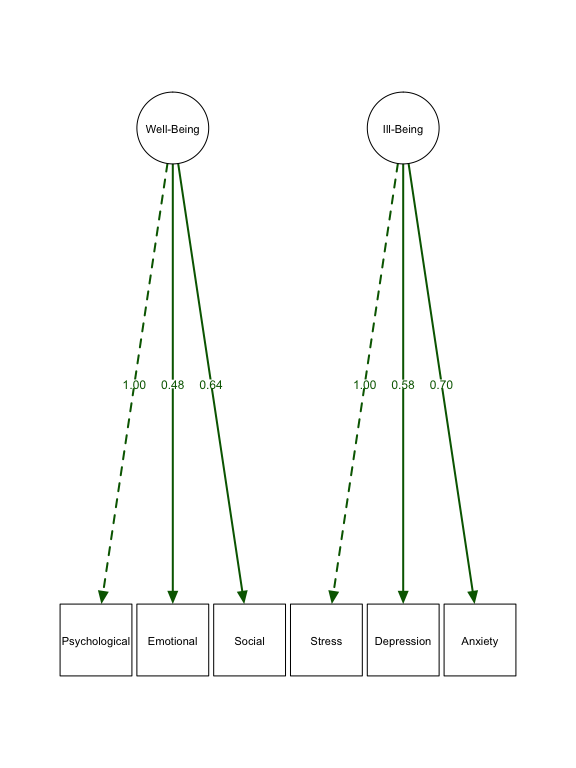


The third model was a bi-factor model, which combined the first two models (see Figure 3). It considers that all of the variables are likely to share a high amount of variance if they all contribute towards global mental health. Thus, it was assumed that all variables would load onto one factor (Mental Health), but that depression, anxiety and stress would also load onto a second factor (ill-being), and psychologial well-being, emotional well-being and social well-being would load onto a different second factor (well-being). As can be seen in Table 1, this model was found to be the best fitting. The CFI and TLI were both above the recommended .90 threshold, SRMR was below the recommended .06 threshold and RMSEA was close to the recommended .08 for a moderate fit.

#### Figure 3.

##### Results of the CFA analysis on model 3.


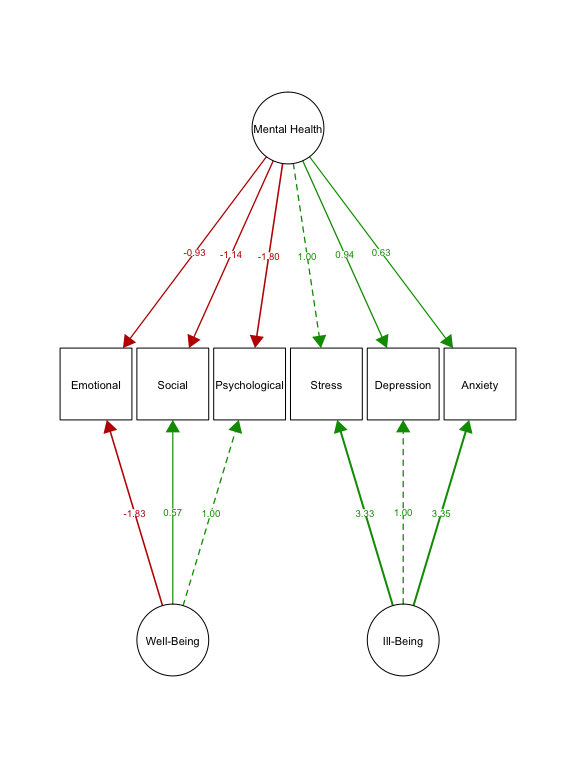


Although the chi-square statistic we obtain from the maximum likelihood statistic is significant for each model, David Kenny states that for models with 75 to 200 cases chi-square is a reasonable measure of fit, but for 400 cases or more it is nearly almost always significant. Thus it is unreliable for big samples like this one.
